# Public views of coronavirus science and scientists: findings from a cross-sectional survey

**DOI:** 10.12688/wellcomeopenres.16780.2

**Published:** 2024-12-05

**Authors:** Rachael Gooberman-Hill, Michelle L. Taylor, Ulrika Maude, Lucy Yardley, Richard Huxtable, Jo Stubbs, Tim J. Peters

**Affiliations:** 1 Elizabeth Blackwell Institute for Health Research, University of Bristol, Royal Fort House, Bristol, BS8 1UH, UK; 2 School of Psychological Science, University of Bristol, Bristol, UK; 3 School of Psychology, University of Southampton, Southampton, UK

**Keywords:** coronavirus, COVID-19, public views, public engagement, uncertainty, trust

## Abstract

**Background:**

Throughout the coronavirus pandemic, references to scientific findings have permeated public-facing communications. Understanding how members of the public view science, scientists and scientific uncertainty should enhance approaches to communication and individuals’ decisions to engage with public health measures, including restrictions and vaccination programmes. This article provides descriptive statistics regarding public views and their univariable associations with key variables: age, gender, ethnicity, keyworker status, shielding status, caring responsibilities, and coronavirus exposure.

**Methods:**

A survey was conducted on our behalf by YouGov in November 2020. The survey asked about: level of public trust in scientists and scientific information; changes in trust between March and November 2020; views about communication of scientific uncertainty; confidence in the accuracy of scientific findings; and views about whether public information accurately represents coronavirus science.

**Results:**

The sample comprised 2,025 individuals in England; 40.5% were ≥55 years old, 51.1% were female; 12.3% identified as members of an ethnic minority/mixed ethnicity. Trust was highest among older respondents and those who identified as of white ethnicity. The concurrent (November 2020) levels of reported trust in scientific information about coronavirus were generally lower than those reported retrospectively for the pandemic’s start (March 2020). There was higher trust and positivity about science among people who had been shielding and among those who had not contracted coronavirus. Around half of respondents did not think that the uncertainty in science was conveyed much or at all, most were confident in the accuracy of coronavirus science, and around half thought that public information was a true representation of the science.

**Conclusions:**

Our study indicates that there is room to improve trust and communication in science. As well as multivariable analyses to explore interrelationships, further research could examine reasons behind change in trust over time and any patterns due to age, ethnicity, and shielding status.

## Introduction

The coronavirus pandemic has caused widespread health, social and economic disruption. Since the start of the pandemic, scientific findings have been published rapidly and the media has provided prolific coverage of science. Public communications about the pandemic have been characterised by frequent references to the rapidly evolving evidence base. Science has been used to explain the spread of the virus and its impact on individuals, society and health systems, as well as to provide evidence about how transmission may be reduced or prevented by individuals’ and society’s actions, such as the use of face coverings. Policy-makers and governments use science to justify decisions about restrictions on society, exemplified most clearly by ‘lockdowns’. Public narratives about vaccines harness rhetoric about the advances made by science, and trust in science may increase confidence in the safety and efficacy of vaccinations. 

The degree of public trust in science and scientists may have implications for the implementation of evidence-based public health measures. Analysis of five recent epidemics highlights the complex relationship between information sources, trust in the information, risk perception, and behaviours ( Majid *et al.*, 2020). Recent analysis of the relationship, since 1970, between trust and global epidemics indicates that young people’s (age 18–25 years) exposure to epidemics does not impact on their views of science but does reduce their trust in scientists ( Eichengreen *et al.*, 2021). In a UK survey conducted in April 2020, over 60% of respondents said that the pandemic had made them *more likely* to listen to expert advice by qualified scientists or researchers ( Open Knowledge Foundation, 2020). The Wellcome Trust’s international ‘Monitor’ survey indicates that public trust is highest in relation to information received from health professionals and academics, whereas trust is lowest for information received from the news and the media generally ( Wellcome Trust, 2018). The Monitor survey also indicates some patterns in public interest and views. For example, women are more interested in health research than men, and older people (≥70 years) are less likely to express high levels of trust in academics compared with younger members of the population ( Wellcome Trust, 2018). In relation to information about coronavirus, the 2020 ‘Monitor’ survey highlighted that people of black and minority ethnicity (BAME) found information about how to act in the coronavirus outbreak less clear than white people and were less likely than white people to trust information conveyed by health sector and government sources ( Wellcome Trust, 2020).

Understanding public views of science requires a consideration of the nature of the information that is being communicated. By default, scientific discoveries comprise a degree of uncertainty, arising from potential errors in measurement, intrinsic biological and/or psycho-social variability, and the issue of statistical precision inherent in sampling from populations. Often, all of these factors are present in studies of human knowledge, attitudes and behaviours. 

Individual and social responses to health and illness take account of uncertainty; during the coronavirus pandemic there has been uncertainty about the illness, its impact, treatment and the ways in which society can cope with or emerge from the pandemic. Tolerance of uncertainty comprises cognitive, emotional, and behavioural responses ( Hillen *et al.*, 2017), and aversion towards uncertainty about the future is well recognised ( Keren & Gerritsen, 1999). Higher individual tolerance of ‘ambiguous uncertainty’ has been associated with greater prosocial behaviours ( Vives & FeldmanHall, 2018). Recent research indicates that some scientists believe that communicating information about scientific uncertainty to members of the public can increase distrust (‘don’t trust much/at all’) and confusion ( Frewer *et al.*, 2003). However, this is strongly countered by the perspective that consideration of public views and the understanding of information, within the context of uncertainty, is needed if science is to become more transparent ( Osman *et al.*, 2018). Importantly, empirical work indicates that ‘epistemic uncertainty’, communicated through facts, numbers and evidence, does not reduce public trust in science or scientists ( Retzbach & Maier, 2015; van der Bles *et al.*, 2020) and that communicating uncertainties does not appear to undermine trustworthiness ( Blastland *et al.*, 2020). Moreover, transparent and honest acknowledgement of uncertainty is arguably an essential part of information that is complete and transparent.

In health and medicine, close attention has been paid to the communication of evidence and uncertainty. In clinical care, shared decision-making is promoted as a means of reaching treatment decisions that are respectful of patients’ informed wishes and use current evidence, including the assessment of future risk and current uncertainties ( Coulter & Collins, 2011). Even in the context of clinical decision-making it is not always clear how best to communicate uncertainty, and insufficient patient involvement may reduce patient satisfaction in such decisions ( Politi *et al.*, 2011). The latest guidance on consent from the UK’s General Medical Council insists that doctors ‘must give patients the information they want or need to make a decision’, which will usually include information regarding ‘uncertainties about the diagnosis or prognosis’. The guidance includes a section on ‘answering questions and dealing with uncertainty’, which states that doctors ‘must answer patients’ questions honestly and accurately’, explaining clearly to the patient any aspects of uncertainty ( General Medical Council, 2020). Although consent to medical treatment differs from the agreement to follow public health guidance, ensuring that members of the public can access accurate scientific information, including aspects of uncertainty, enables a higher degree of informed decision-making and counters misinformation. Although some measures in the pandemic (such as ‘lockdown’ or ‘stay at home’ restrictions) have been mandatory and could not have been avoided lawfully by individuals, others have been premised on recommendations (e.g. hand washing, vaccination). In either case, however, individuals make decisions either to adhere or not, and since public health guidance during the COVID-19 pandemic has been represented as premised on ‘the science’, the communication of uncertainty contained in science is an important part of transparency and the facilitation of informed decisions.

Understanding how members of the public view science and its uncertainties in a pandemic helps to improve engagement and agreement between the public, scientists and producers of guidance. In the context of medical decision-making, clear and mutual understanding about degrees of certainty in evidence is a key part of achieving agreement about next steps in treatment or actions. A similar principle can apply in relation to public health measures, in which shared understanding and trust can form the bedrock of agreement about the appropriateness of a course of action, including behaviours that reduce or increase virus transmission, decisions about treatments, provision of health care and confidence in and uptake of vaccines. This applies even when measures are mandatory, since agreement that these measures are necessary and that they are based on appropriate evidence is key to adherence.

This article describes data from a survey of public views about coronavirus science and scientists in light of factors that might be associated with those views. The study aimed to provide descriptive statistics regarding these public views and their univariable associations with key individual characteristics. Guided by previous research and discussions within the multidisciplinary research team, we selected seven characteristics of potential relevance. Three were drawn from the literature described above (age, gender, and ethnicity) and four were identified by the team (keyworker status, ‘shielding’ (quarantining), carer status, and whether a person has been diagnosed with or believes that they have had COVID-19). These data were intended as an exploratory investigation, with no pre-specified hypotheses about the likely direction or magnitude of the relationships. The article provides descriptive statistics from the study, identifies noteworthy findings, and provides access to the data.

## Methods

In 2020, we commissioned the market research company YouGov to conduct a cross-sectional survey of public views of coronavirus science in relation to the COVID-19 pandemic. Respondents were recruited by YouGov Plc UK from their panel of over 800,000 adults in the UK, with the aim of achieving a minimum sample size of 2000 participants. Potential participants were already members of the YouGov panel and were contacted by YouGov through an alert via the YouGov online portal. If a potential participant was interested in taking part, the portal directed them to a study-specific consent statement about the study, which asked them to confirm that they have read the information provided, that participation was voluntary, they once completed they are unable to withdraw and that they agree to take part. After accepting these statements by way of consent, participants were able to complete the survey online via the YouGov portal. Sample sizes of 2000 are a standard one-day poll size for YouGov surveys that aim for a margin of error (half-width of a 95% confidence interval (CI)) of 2–3 percentage points around point estimates of proportions. Here these criteria would hold for reasonably sized subgroups – that is, at least 20% of the total depending on the magnitude of the proportion estimated. We also conducted power analyses (using G*Power) to ensure that this sample size would facilitate future logistic regression modelling (not reported here) of up to 10 characteristics, assuming small-medium effect sizes (odds ratios >1.6) across a range of potential distributions of responses, a two-sided 5% significance level and with a minimum of 80% power. Participants were eligible if they were aged ≥18 years and lived in England. Sampling was conducted using interlocking quotas on respondent numbers in terms of age, gender, ethnicity, region, education level, and social grade to achieve a broadly representative sample of the adult English population. Ethnicity reporting followed the recommended format of the ‘Harmonized country specific ethnic group question’ for England ( Office for National Statistics, 2015). Social grade was identified by YouGov in accordance with National Readerships (NRS) Social Grades, a classification based on occupation. In this survey, NRS Social Grades are grouped into two categories, ABC1 (managerial, supervisory) and C2DE (manual and casual workers, pensioners and unemployed people). We stipulated ‘one response per household’ in our survey design to YouGov, and data were cross-sectional, collected from each individual on a single day.

Data collection took place from 5 ^th^-13 ^th^ November 2020; Thursday 5 ^th^ November was the first day of the second ‘lockdown’ in England when the Health Protection Regulations (Coronavirus restrictions) came into effect for 28 days (( Health Protection (Coronavirus, Restrictions) (England) Regulations, 2020c; SI 1200)). This period of lockdown included limitations on movement, including a restriction on leaving home without ‘reasonable excuse’, and a limit of two people gathering in an indoor space or a public outdoor space. Preceding this, the first English lockdown came into effect on 26 ^th^ March 2020 with the Lockdown Regulations ( Health Protection (Coronavirus, Restrictions) (England) Regulations, 2020a; SI 350); these were replaced on 4 ^th^ July by Health Protection (Coronavirus restrictions), which continued into and beyond the second lockdown ( Health Protection (Coronavirus, Restrictions) (England) Regulations, 2020b; SI 684). 

The study was designed with input from the University of Bristol Elizabeth Blackwell Institute for Health Research’s Public Advisory Group, who discussed the study questionnaire and contributed towards its development. The Advisory Group also provided insights into the information sources that they used, which helped to shape the design of the study. The study was approved by the Research Ethics Committee of the Faculty of Health Sciences, University of Bristol (ref: 108683) and consent processes are described above.

Personal characteristics were collected by YouGov as part of their standard dataset in advance of our survey, including gender, age, ethnicity, employment status, parental status and marital status. As part of our survey we included additional, standard YouGov questions such as house type, household size, number of children in household, and educational level. Our survey asked participants about their status during the COVID-19 pandemic, including whether they considered themselves a ‘keyworker’, ‘carer’, ‘shielding’, and had had exposure to COVID-19 (both diagnosed and assumed). ‘Keyworkers’ were those in job roles essential and critical to the COVID-19 response, and followed the categories of such workers listed by the Government including those employed in health and social care, education and childcare, key public services, local and national government, food and other necessary goods, public safety and national security, transport and utilities, communication and financial services. ‘Shielding’ essentially amounts to quarantining: the Government and/or NHS advised particular groups of vulnerable persons not to leave their homes at any time and to avoid all face-to-face contact with people outside their household during the periods of lockdown.

Participants were also asked questions about their views of science, including trust in science and scientists, the accuracy of science, how scientific uncertainty was communicated, and whether public information provided a correct representation of science. In questions about trust in science and scientists, participants were asked to reflect on their views in March 2020, before the start of the first lockdown restrictions in England, and to define their current views in November 2020, at the start of the second lockdown restrictions in England. Questions were phrased using the term ‘coronavirus’ to accord with most publicly available information and maximise the coherence of the questionnaire. Responses were offered across five options – ‘trusted a lot’, ‘trusted a little’, ‘didn’t trust very much’, ‘didn’t trust at all’, ‘don’t know’ – with some variation in wording depending on the question. The questions and response options are provided in the results section.

From the survey data, we derived descriptive statistics of responses to questions on views of science across the key personal characteristics of respondents. To do so we used YouGov’s platform CRUNCH.io 2021 as well as Windows 10 Microsoft Office 365, Excel with standard settings. Although these descriptive statistics do not take account of any associations between the variables, they provide an immediate insight into patterns in views of science and the coronavirus pandemic across groups of respondents. YouGov stipulate a minimum sample size of 50 for calculating statistically valid summary statistics across groups of respondents. While this does result in some loss of granularity, we combined groups of respondents where necessary, for example in terms of age, ethnicity, education level, employment status, household size, house type lived in, parental status, marital status, shielding status, and coronavirus exposure. We also removed respondents who answered these key characteristics as ‘don’t know’ or ‘prefer not to say’, resulting in fewer than 2000 respondents for some characteristics. Details of how categories were combined are given in the footnotes of each table. When asking about coronavirus exposure, rather than the confirmed infection status of individuals, we were primarily interested in whether people believed they had had coronavirus, irrespective of whether they had tested positive. Therefore, we combined the answers of respondents who identified themselves as ‘I think I have had coronavirus but have not been tested’ with those who identified themselves as having tested positive for coronavirus. Similarly, respondents who had believed themselves to have had coronavirus but had tested negative, remained as a separate group from those who simply identified themselves as ‘I haven’t had coronavirus’. Finally, we combined responses of ‘didn’t trust much’ and ‘didn’t trust at all’ to obtain adequate sample sizes.

## Results

### Sample

The total sample size was 2025 respondents for age, gender, region, social grade, employment status, and parental status. Sample sizes were smaller for ethnicity (N = 1996), marital status (N = 2009), education level (N = 1930), household size (N = 1962), house type (N = 1949), number of children in household (N = 1947), keyworker status (N = 1945), carer status (N = 1972), shielding status (N = 1986), and coronavirus exposure (N = 1791) due to missing data. All percentages have been calculated from their respective total non-missing sample sizes. Sample information is shown in Table 1; summary statistics are available as extended data available through the repository as described in the data availability statement ( Gooberman-Hill *et al.*, 2021b).

**Table 1.** Sample sizes, point estimates, and precision of key characteristics of respondents.

Key characteristics	Sub-categories	Respondents	N	%	Margin of error %	Lower CI	Upper CI
Age	18–34 years	549	2025	27.1	1.94	25.2	29.1
	35–54 years	655	2025	32.3	2.04	30.3	34.4
	>55 years	821	2025	40.5	2.14	38.4	42.7
Gender	Male	990	2025	48.9	2.18	46.7	51.1
	Female	1035	2025	51.1	2.18	48.9	53.3
Ethnicity	White	1750	1996	87.7	1.44	86.2	89.0
	Ethnic minority	246	1996	12.3	1.44	11.0	13.8
Keyworker status	Yes	488	1945	25.1	1.93	23.2	27.1
	No	1458	1945	75.0	1.92	73.0	76.8
Carer status	Yes – current	207	1972	10.5	1.36	9.2	11.9
	Yes – past	303	1972	15.4	1.59	13.8	17.0
	No	1462	1972	74.1	1.93	72.2	76.0
*Shielding status	Yes/household	319	2001	15.9	1.61	14.4	17.6
	No	1682	2001	84.1	1.61	82.4	85.6
Coronavirus exposure	No, haven’t had it	1192	1791	66.6	2.18	64.3	68.7
	I thought I had it, but tested negative	319	1791	17.8	1.77	16.1	19.7
	Yes (tested positive, not tested)	279	1791	15.6	1.68	14.0	17.3

Note: YouGov Data; Total sample size was 2,025 adults. Fieldwork was undertaken between 5th - 13th November 2020. The survey was carried out online. The figures have been weighted and are representative of all English adults (aged 18+).*respondents were able to tick more than one option for ‘Shielding status’, thus, vote counts tally to more than the weighted N of 1986 calculated from the interlocking quotas. Margins of error and 95% Confidence Intervals calculated using modified Wald method ( Agresti & Coull, 1998).

The figures have been weighted to provide a representative sample of English adults by age, gender, ethnicity, region, education level, and social grade. Within our sample, respondents aged ≥55 years constituted the largest group (40.5%), with those aged 35–54 years the second largest (32.3%), and those aged 18–34-years the smallest group (27.1%). There were slightly more females (51.1%) than males (48.9%). Respondents who identified themselves as white (i.e., English/Welsh/Scottish/Northern Irish/British, Irish, any other white) were the largest group (87.7%), with those identifying as an ethnic minority, including mixed ethnicity, accounting for 12.3%. The majority of respondents were not currently keyworkers (75.0%), carers (74.1%), or shielding (84.7%), and two-thirds did not believe that they had had coronavirus (66.6%). Each of the ABC1 and C2DE social grade groups comprised about half the sample (58.0% and 42.0% respectively); broadly ABC1 comprises managerial, administrative, professional roles and C2DE comprises manual workers, state pensioners, casual workers and unemployed people with state benefits only.

### Trust in scientific information about coronavirus

Respondents were asked: ‘ *Thinking specifically about publicly available information (e.g. information from the Government, news outlets etc.), at the start of the Coronavirus outbreak in England (i.e. in March 2020)...Overall, to what extent, if at all, did you trust the scientific information available on Coronavirus at that time?’*. Response options were *‘trusted a lot*’, ‘ *trusted a little*’, ‘ *didn’t trust very much*’, ‘ *didn’t trust at all*’, ‘ *don’t know*’.

When describing the views that they held in March 2020, the majority of the 2025 respondents trusted ‘a lot’ (37.2%, 95% CI 35.1% to 39.4%) or ‘a little’ (37.6%, 95% CI 35.5% to 39.7%), while substantially fewer people ‘didn’t trust much/at all’ (19.0%, 95% CI 17.3% to 20.7%) or ‘didn’t know’ (6.3%, 95% CI 5.3% to 7.4%).

AGE: trust was highest in the oldest age group (47.0% of ≥55 years answered ‘a lot’ compared with 25.3% amongst 18–34-year-olds). However, the youngest age group had the highest levels of ‘trust a little’ and ‘didn’t trust much/at all’. The percentage of respondents answering ‘don’t know’ was around five times higher in the youngest compared with the oldest age group.

GENDER: slightly more females than males expressed trust in the scientific information available on coronavirus in March 2020 (76.7% trusted ‘a lot’ or ‘a little’ versus 72.9%, respectively), while more males answered ‘didn’t trust much/at all’ or ‘don’t know’. The differences between the genders in opinions on trust were 2–3 percentage points across all answers. 

ETHNICITY: trust in coronavirus science in March 2020 was highest in white respondents (39.5% answered ‘trusted a lot’), while trust was lowest amongst ethnic minorities (22.4% ‘didn’t trust much/at all). Ethnic minorities were also three times more likely to answer ‘Don’t know’.

KEYWORKER STATUS: more non-keyworkers than keyworkers trusted science ‘a lot’ in March, but also more often reported ‘didn’t trust much/at all’’. More keyworkers trusted ‘a little’ and answered ‘don’t know’. Differences across groups were, however, all under 5 percentage points.

CARER STATUS: respondents who had been carers in the past showed the highest levels of ‘trust a lot’ (40.6%) and ‘didn’t trust much/at all’ (21.8%), whereas those who had never been carers had the highest levels of ‘trust a little’ and ‘don’t know’. Differences between all the groups across all answers were between 1–4 percentage points.

SHIELDING STATUS: respondents who were not shielding were the most likely to answer ‘trust a lot’ or ‘trust a little’, while those shielding were most likely to answer ‘didn’t trust much/at all’. Again, the differences between the groups across all answers were small, at between 1–5 percentage points.

CORONAVIRUS EXPOSURE: respondents who tested negative were the most likely to answer ‘trust a lot’ (42.0%), while those who had tested positive/thought they had had coronavirus, but had not been tested, were the most likely to answer ‘didn’t trust much/at all’ (26.5%), which is almost 13 percentage points higher than those who thought they had had coronavirus, but had tested negative. 

To assess trust in scientific information in November 2020, respondents were asked: *“Still thinking specifically about publicly available information (e.g. information from the Government, news outlets etc.)...To what extent, if at all, do you trust the scientific information available on Coronavirus now?”* Response options were as above.

When describing their current views, in November 2020, overall trust in coronavirus science had fallen, with substantially fewer people answering ‘trust a lot (25.4%, 95% CI 23.6% to 27.4%), marginally more people answering ‘trust a little’ (42.0%, 95% CI 39.8% to 44.1%), and substantially more people answering ‘don’t trust much/at all’ (26.1%, 95% CI 24.2% to 28.0%) compared with March 2020. There was no change in those answering ‘don’t know’ (6.5%, 95% CI 5.5% to 7.7%). 

AGE: in November 2020, trust in scientific information about coronavirus was still higher in the oldest age group; however, the difference between trust in the oldest and youngest age groups had reduced from 22 to 9 percentage points since March. This decline was largely through a shift to ‘trust a little’ (in those aged ≥55 years), and an increase in ‘don’t trust much/at all’ of 4–9 percentage points across all ages, with around one in four people in November in all age groups reporting ‘don’t trust much/at all’. There were no changes in ‘don’t know’ across ages between March and November.

GENDER: all of the patterns across males and females observed in March pertained in November, and again the disparity between males and females was small, at about 3–4 percentage points.

ETHNICITY: the patterns observed in March were broadly consistent with those in November 2020, but fewer respondents of all ethnicities answered ‘trust a lot’ than in March (25.5%, down from 37.2%). This decline appeared to be driven by a shift to ‘trust a little’ in white respondents (42.3%, up from 37.5%), and a shift to ‘don’t trust much/at all’ in all ethnicities (increase of around 7 percentage points in November compared with March 2020).

KEYWORKER STATUS: patterns of relative trust across keyworkers and non-keyworkers were broadly similar to those in March, albeit with a notable decrease in ‘trust a lot’ in all respondents (from 39.1% to 26.2% in non-keyworkers) and increase in ‘don’t trust much/at all’ in all respondents (from 19.2% to 27.2% in non-keyworkers).

CARER STATUS: trust in scientific information about coronavirus in November had shifted from ‘trust a lot’ to ‘trust a little’ across all carer groups, compared with March, although differences across groups were small at 1–2 percentage points. Those who had been carers in the past maintained the highest levels of ‘don’t trust much/at all’, and at least 1 in 4 respondents across all groups reported ‘don’t trust much/at all’. 

SHIELDING STATUS: those who were shielding had slightly higher trust in scientific information than those who were not shielding. This apparent ‘switch’ from March 2020 was driven mainly by those who were not shielding shifting their opinion from ‘trust a lot’ to ‘don’t trust much/at all’ between March and November (increase of around 8 percentage points), while those who were shielding shifted their opinion to ‘trust a little’ (increase of about 6 percentage points).

CORONAVIRUS EXPOSURE: patterns of trust in scientific information in November were broadly similar across groups to those in March, but those who had tested positive/thought they had had coronavirus, but had not been tested, showed the largest decline in ‘trust a lot’ (around 15 percentage points) and the highest ‘don’t trust much/at all’ at 32.3%. Those who tested negative or who thought that they had never had coronavirus showed the largest increases in ‘don’t trust much/at all’ (around 8 percentage points).

### Trust in scientists to conduct accurate and reliable research

Respondents were asked: *“Thinking back to BEFORE the Coronavirus outbreak in England (i.e. before March 2020)...Overall, to what extent, if at all, did you trust UK scientists to, as far as possible, conduct accurate and reliable research?”*. Response options were: ‘ *trust a lot*’, ‘ *trust a little*’, ‘ *don’t trust very much*’, ‘ *don’t trust at all*’, ‘ *don’t know*’.

 In March 2020, trust in scientists was high overall, with the majority of respondents answering ‘trust a lot’ (48.3%, 95% CI 46.1% to 50.5%) or ‘trust a little’ (31.8%, 95% CI 29.8% to 33.9%), while substantially fewer people answered ‘didn’t trust much/at all’ (12.3%, 95% CI 11.0% to 13.9%) or ‘don’t know’ (7.6%, 95% CI 6.5% to 8.8%). Overall, trust was higher in scientists than in coronavirus science, with 80.1% (95% CI 78.3% to 81.8%) answering ‘trusted a lot’ or ‘a little’, versus 74.8% (95% CI 72.9% to 76.7%), respectively.

AGE: the highest levels of trust were observed in the oldest (≥55 years) age group (53.8%), with the youngest (18–34 years) age group showing the lowest levels of ‘trust a lot’ (39.2%). Levels of trust were comparable across age groups in those who answered ‘trust a little’ and ‘didn’t trust much/at all’; however, there was an 8 percentage point difference between the youngest age and oldest age groups of those answering ‘don’t know’.

GENDER: females showed slightly higher levels of trust than males in March 2020 (49.5% ‘trust a lot’ and 32.0% ‘trust a little’), while males showed slightly higher levels of ‘didn’t trust much/at all’ in scientists (13.2%) and ‘don’t know’ (8.2%). Again, there was only a difference of 1–2 percentage points between genders across all responses in March 2020.

ETHNICITY: the highest levels of trust were in white respondents, with 51.4% answering ‘trust a lot’, compared with 26.8% of ethnic minorities, who were more likely to answer ‘trust a little’. The percentages of ethnic minorities answering ‘didn’t trust much/at all’ and ‘don’t know’ were around twice that of white respondents.

KEYWORKER STATUS: non-keyworkers expressed higher trust in scientists than keyworkers (50.1% versus 45.9% for ‘trust a lot’). Although keyworkers expressed marginally higher ‘trust a little, ‘didn’t trust much/at all’, and ‘don’t know’ in science, these were all within 1–2 percentage points of non-keyworkers.

CARER STATUS: those who were current carers had the highest response rate for ‘trust a lot’ (50.2%), but there was only a 3 percentage point difference in trust between all carer groups. However, those who had been carers in the past showed higher levels of ‘didn’t trust much/at all’ than the other two groups.

SHIELDING STATUS: respondents who were shielding or not shielding were most likely to answer ‘trust a lot’, but those who were not shielding were more likely to answer ‘trust a lot’ than those who were shielding (49.7%), while those who were shielding were the most likely to answer ‘didn’t trust much/at all’ (16.0%).

CORONAVIRUS EXPOSURE: respondents who tested negative were more likely and the most likely to answer trust ‘a lot’ (54.5%), while those who had tested positive/thought they had had coronavirus, but not been tested were the most likely to answer ‘didn’t trust much/at all’ (14.3%).

Respondents were also asked: *“Now thinking about the current day...Overall, to what extent, if at all, do you trust UK scientists to, as far as possible, conduct accurate and reliable research?”* Response options were as above.

By November 2020, overall trust in scientists had declined, with substantially fewer respondents answering ‘trust a lot’ (40%, 95% CI 37.9% to 42.2%), marginally more answering ‘trust a little’ (36.0%, 95% CI 34.0% to 38.2%), and substantially more people answering ‘don’t trust much/at all’ (17.6%, 95% CI 16.0% to 19.4%) compared with March 2020. There was no real change in respondents answering ‘don’t know’ (6.4%, 95% CI 5.4% to 7.4%). 

AGE: trust in scientists declined across all age groups, with the biggest decline in the oldest age group (from 53.8% to 43%). There was an increase in ‘don’t trust much/at all’ across all age groups, compared with March, with the biggest increase in those aged 34–54-years old (from 11.9% to 19.1%). The youngest respondents still had the highest levels of ‘don’t know’.

GENDER: although trust declined overall, patterns of opinions across males and females followed those in March, with differences of only up to 3 percentage points across all responses.

ETHNICITY: patterns of trust in November were broadly consistent with those in March; however, there was an overall decline in ’trust a lot’ (from 48.4% to 40.1%) as respondents of all ethnicities shifted to ‘trust a little’ and ‘don’t trust much/at all’, with white respondents showing the largest shift to ‘don’t trust much/at all’ (increase of about 6 percentage points), while ethnic minorities showed 2 percentage point increases in ‘trust a little’ and ‘don’t trust much/at all’. The percentage of ethnic minorities answering ‘don’t know’ was around three times that of white respondents. 

KEYWORKER STATUS: fewer keyworkers and non-keyworkers answered ‘trust a lot’ in November than in March, and the decline in trust in both groups was due more to a larger increase in ‘don’t trust much/at all’ (5–6 percentage points) than a shift to ‘trust a little’ (3–5 percentage points). More keyworkers answered ‘don’t know’ than non-keyworkers.

CARER STATUS: all groups of carers and non-carers showed declining levels of ‘trust a lot’ in November compared with March 2020. Some of this change was due to a shift to ‘trust a little’ (increase of 4–6 percentage points), and also due to an increase in ‘didn’t trust much/at all’ across all groups (3–6 percentage points). There were no notable differences in ‘don’t know’ answers, either among groups, or between March and November. 

SHIELDING STATUS: while the same broad patterns of trust from March pertained in November, trust declined overall, with a shift towards ‘trust a little’ (overall increase of 3 percentage points) and an increase in ‘don’t trust much/at all’ in both groups (overall 5 percentage points). Those who were not shielding showed the greatest increase in ‘don’t trust much/at all’ (from 11.5% to 17.5% between March and November).

CORONAVIRUS EXPOSURE: patterns of trust remained broadly the same as in March, with a decline in ‘trust a lot’, driven by a shift towards ‘trust a little’, and ‘don’t trust much/at all’ in all groups. Those who had tested negative showed the largest increase in ‘trust a little’ (from 28.8% to 37.6%), and those who did not think they had had coronavirus showed the largest increase in ‘don’t trust much/at all’ (from 11.6% to 17.4%). 

### Communicating the uncertainty in coronavirus science

Respondents were asked: *“Please read the following information before answering the question below...Scientific observations must take into account both accuracy and precision. The best scientific observations are both accurate and precise, but it is rare for observations to be 100% accurate and precise. This means there is always some level of uncertainty in scientific observations. When science is then used to make recommendations to the public, it must be considered that this uncertainty remains. To what extent, if at all, do you think that the potential uncertainty in coronavirus science is communicated in the information you receive?”*. Response options were: ‘ *a lot*’, ‘ *a fair amount*’, ‘ *not very much*’, ‘ *not at all*’, ‘ *don’t know*’.

Overall, in November 2020, approximately equal numbers of respondents thought that uncertainty was communicated ‘a lot/a fair amount’ (46.2%, 95% CI 44.0% to 48.3%) as felt that it had been communicated ‘not very much/not at all’ (43.3%, 95% CI 41.2% to 45.5%), with 10.5% (95% CI 9.3% to 11.9%) of respondents answering ‘don’t know’.

AGE: the oldest respondents (≥55 years old) were the most likely to answer that uncertainty was communicated both ‘a lot/a fair amount’ and ‘not very much/not at all’. The percentages of younger age groups (18–34 and 35–49 year olds) answering ‘don’t know’ were around twice that of the oldest age groups. 

GENDER: more females than males agreed that uncertainty had been communicated ‘a lot/a fair amount’, while more males answered ‘don’t know’; however, the differences in all responses across genders were within 3 percentage points.

ETHNICITY: respondents from ethnic minorities were the most likely to agree that uncertainty was communicated ‘a lot/a fair amount’, while white respondents were more likely think that uncertainty had been communicated ’not very much/not at all’. The disparity between those who did and did not think uncertainty was communicated to them was greater in ethnic minorities than in white respondents (13 versus 1 percentage point, respectively). More ethnic minorities than white respondents answered ‘don’t know’.

KEYWORKER STATUS: more non-keyworkers than keyworkers agreed that uncertainty had been communicated ‘a lot/a fair amount’ (47.3% versus 44.9%, respectively), and vice versa for ‘not much/not at all’ (44.3% versus 43.8%, respectively). More keyworkers responded ‘don’t know’ than non-keyworkers; however, the difference was within 2 percentage points.

CARER STATUS: respondents who had been carers in the past were the most likely to answer ‘a lot/a fair amount’ (52.8%), while those who had never been carers were the most likely to answer ‘not much/not at all’ (44.7%), and ‘don’t know’ (10.0%).

SHIELDING STATUS: those who were shielding were the most likely to answer that uncertainty had been communicated ‘a lot/a fair amount’, but there was less than a 2 percentage point difference between those of different shielding status in their views about the degree to which uncertainty had been communicated.

CORONAVIRUS EXPOSURE: respondents who did not believe they had had coronavirus, or believed they had had it, but tested negative, were the most likely to agree that uncertainty had been communicated ‘a lot/a fair amount’. While those who had tested positive for coronavirus/believed they had had coronavirus, but had not been tested, were the most likely to answer that uncertainty in the science was ‘not much/not at all’ communicated. There was only a 2–5 percentage point difference across all the groups in their answers.

### Confidence in the accuracy of coronavirus science

Respondents were asked: ‘ *As a reminder, by 'Coronavirus science', we mean science that seeks to: Understand and predict the progress of the Coronavirus outbreak; Understand the virus itself; Prevent the spread of the virus; Develop treatments and vaccines for the virus. Overall, how confident, if at all, are you that ‘Coronavirus science' is accurate?’*. Response options were: ‘ *very confident*’, ‘ *fairly confident*’, ‘ *not very confident*’, ‘ *not at all confident*’ and ‘ *don’t know*’.

Overall, the majority of respondents were ‘fairly confident’ in the accuracy of coronavirus science (50.1%, 95% CI 47.9% to 52.3%), with about one third answering ‘not very/not at all confident’ (32.1%, 95% CI 30.1% to 34.2%), only 10.0% (95% CI 8.7% to 11.4%) being ‘very confident’, and 7.8% (95% CI 6.7% to 9.0%) answering ‘don’t know’.

AGE: the youngest respondents had the most confidence in the accuracy of science (12.0% ‘very confident’), while older respondents had higher levels of ‘fairly confident’. The oldest respondents had the highest number of ‘not very/not at all’ confident (33.3%). The percentage of respondents answering ‘don’t know’ was almost 3 times higher in the youngest age group than in the oldest age group.

GENDER: more females than males answered ‘very confident’, ‘fairly confident’, and ‘not very/not at all confident’, while more males than females answered ‘don’t know’ (8.9% versus 6.7%). However, there was only a 1–2 percentage point difference between the genders across these responses.

ETHNICITY: the highest number of ‘very confident’ and ‘fairly confident’ answers were from white respondents, while the highest number of ‘not very/not at all confident’ answers were from ethnic minority respondents. The greatest difference between the ethnic groups was in those who answered ‘fairly confident’ (51.6% white versus 41.5% ethnic minorities). The percentage of respondents belonging to ethnic minorities who answered ‘don’t know’ was more than twice that of white respondents.

KEYWORKER STATUS: more keyworkers than non-keyworkers answered ‘very confident’ and ‘not very/not at all confident’, and ‘don’t know’, while more non-keyworkers than keyworkers were ‘fairly confident’ in the accuracy of coronavirus science. There was a 1–5 percentage point difference between the groups across all responses.

CARER STATUS: those who had been carers in the past had the highest percentages of ‘very confident’, ‘fairly confident’, and ‘not very/not at all confident’. There was a 1–3 percentage point difference between the groups across these three responses. Current carers were the most likely to answer ‘don’t know’, with a 5 percentage point difference between all groups.

SHIELDING STATUS: respondents who were shielding (or who had someone in their household shielding) were the most likely to answer ‘very confident’ (11.9%) ‘or ‘fairly confident’ (55.2%) in the accuracy of coronavirus science, while those not shielding were the most likely to answer ‘not very/not at all confident’ (33.0%) and ‘don’t know’ (7.1%).

CORONAVIRUS EXPOSURE: respondents who believed they had had coronavirus, but had tested negative, were the most likely to answer ‘very confident’ (13.2%) and ‘fairly confident’ (57.7%). Those who had tested positive/believed they had had coronavirus, but had not been tested, were the most likely to answer ‘not very/not at all confident’ (37.6%). The greatest differences in opinion were between those who had tested negative and those who had tested positive/believed they had had coronavirus, but had not been tested, with a 13 percentage point difference in those who were ‘fairly confident’, and a 14 percentage point difference in those were ‘not very/not at all’ confident.

### Is the public information a true representation of science?

Respondents were asked: ‘ *To what extent do you agree or disagree with the following statement? - The public information that is accessible (e.g. about the outbreak, how to prevent the spread of the virus, the development of treatments and vaccines etc.) is a true representation of 'Coronavirus science'’*. Response options were: ‘ *strongly agree*’, ‘ *tend to agree*’, ‘ *tend to disagree*’, ‘ *strongly disagree*’ and ‘ *don’t know*’

Overall, the majority of respondents ‘tended to agree’ that publicly available information was a true representation of coronavirus science (51.3%, 95% CI 49.1% to 53.4%), while about one quarter ‘tended to disagree/strongly disagree’ (25.4%, 95% CI 23.5% to 27.3%), while only 10.1% (95% CI 8.8% to 11.5%) of respondents ‘strongly agreed’, and 13.3% (95% CI 11.9% to 14.9%) answered ‘don’t know’.

AGE: the oldest respondents were the most likely to answer ‘strongly agree’ and ‘tend to agree’ (11.2% and 57.5%, respectively), while the youngest respondents answered ‘tend to disagree/strongly disagree’ (29.9%). The percentage of the youngest respondents answering ‘don’t know’ was around twice that of the oldest respondents. 

GENDER: more males than females answered ‘strongly agree’ or ‘tend to agree’ (11.2% and 52.5%, respectively), while more females answered ‘tend to disagree/strongly disagree’ (27.5%). There was a 1–4 percentage point difference between males and females across all responses.

ETHNICITY: white respondents were the most likely to ‘strongly agree’ or ‘tend to agree’ (10.6% and 52.2%, respectively), while respondents from ethnic minorities were the most likely to answer ‘tend to disagree/strongly disagree’ (27.2%). There was a 5 percentage point difference between ethnic minorities and white respondents who answered ‘don’t know’.

KEYWORKER STATUS: there was no difference between keyworkers and non-keyworkers who answered ‘strongly agree’, but more non-keyworkers answered ‘tend to agree’ (53.6%), while more keyworkers answered ‘tend to disagree/strongly disagree’ (29.5%). There was a 1 percentage point difference between different groups in those who answered ‘don’t know'.

CARER STATUS: respondents who were current or past carers were the most likely to ‘strongly agree’ or ‘tend to agree’ that public information was a true representation of science (around 10.0% and 57.0%, respectively), while respondents who had never been carers were the most likely to answer ‘tend to disagree/strongly disagree’, or ‘don’t know’ (26.1% and 13.7%, respectively).

SHIELDING STATUS: respondents who were shielding, or who had someone in their household who was shielding, were the most likely to ‘strongly agree’ and ‘tend to agree’ that public information was a true representation of science (14.1% and 54.5%, respectively), while respondents who were not shielding were the most likely to answer ‘tend to disagree/strongly disagree’, or ‘don’t know’ (25.8% and 13.2%, respectively).

CORONAVIRUS EXPOSURE: respondents who believed they had had coronavirus, but tested negative, were the most likely to answer ‘strongly’ agree or ‘tend to agree’ that public information was a true representation of coronavirus science, while those who had tested positive for coronavirus/believed they had had coronavirus, but had not been tested, were the most likely to answer ‘tend to disagree/strongly disagree’ or ‘don’t know’ (34.1% and 13.3%, respectively). Again, there were large differences in opinion between those who had tested negative and those who had tested positive/believed they had had coronavirus, but had not been tested (14 percentage points for those who ‘tend to agree’ and 13 percentage points for those who ‘tend to disagree/strongly disagree’).

## Discussion

We presented descriptive statistics of public views about coronavirus science in relation to age, gender, ethnicity, keyworker status, carer status, shielding, and coronavirus exposure to gain insight into how different groups of people feel about science, scientists and the communication of science relating to the coronavirus pandemic. The data showed some patterns in views of science which might, to an extent, underpin responses to public health measures used to manage the pandemic. We discuss below those findings that present challenges for the role of science in guiding public policy, and also some observations unique to the coronavirus pandemic.

One of the most concerning findings was the difference in levels of trust in coronavirus science in March and November 2020, with a decline in the proportion of people responding that they trusted coronavirus science ‘a lot’ and an increase in those saying that they ‘didn’t trust much/at all’. Similarly, trust in scientists to conduct accurate and reliable research decreased between March and November 2020, albeit to a lesser extent. Notwithstanding the potential for recollection biases, whereby people were asked to recall their feelings from months previously, these changes indicate an overall reduction in trust in science over the first eight and a half months of the pandemic. The most notable differences in trust occurred in people of different ages and ethnicities. People who were older expressed higher levels of ‘trust a lot’ than other age groups, as did those who were white.

The finding that people of white ethnicity were more likely to express higher trust in scientific information than people of ethnic minority backgrounds aligns with findings of the Wellcome Monitor Survey, 2020, in which members of the BAME group expressed lower levels of trust in information from health sector and government sources than people who identified as white. Differences in trust by age were not found in the Wellcome Monitor, and so our findings are worthy of further investigation. 

Some of the most interesting changes in trust occurred in people of different health status. The highest levels of ‘trust a lot’ were in respondents who thought they had had coronavirus, but had tested negative. When reflecting on their views about coronavirus science in March 2020, those who were not shielding expressed the highest levels of ‘trust a lot’ or ‘trust a little’, while those who had been shielding (or who had someone in their household shielding) expressed the highest levels of ‘didn’t trust much/at all’. By November 2020, this pattern was reversed, such that those who were shielding were slightly more likely to trust coronavirus science ‘a lot’ or ‘a little’ (although levels of ‘trust a lot’ declined in both groups across time). This change in relative trust was largely driven by the change in the opinions of those who were not shielding, perhaps as a consequence of the nature and application of restrictions on those who believed themselves to be less at risk of coronavirus. We note that this ‘switch’ in opinion was only observed in trust in coronavirus science, rather than in scientists in general to conduct accurate and reliable research, and that people who were not shielding were also less likely to be ‘very confident’ or ‘fairly confident’ in the accuracy of coronavirus science, as well as less likely to ‘strongly agree’ or ‘tend to agree’ in November 2020 that public information was a true representation of coronavirus science. This distinction between opinions of those with different health statuses is worthy of further investigation. Perhaps surprisingly, we did not find substantial differences in relation to gender, keyworker, or carer status, although there were some consistent patterns across these questions.

Views about the communication of uncertainty and about confidence in accuracy provide insight into the detail that may underpin trust in science and scientists. Just under half of respondents thought that uncertainty was communicated ‘not very much/not at all’. Early presentations of research during the pandemic, for instance, government briefings, did not routinely include portrayals of uncertainty, such as the very wide confidence intervals around the predicted number of cases, hospital admissions, or deaths. For this study, we collected data in November 2020, nearly eight months after the first restrictions came into action in the UK, at a time when some of these metrics had begun to appear. This might account for the apparently equal division between those who did and did not think uncertainty had been communicated. Around 60% of respondents were ‘fairly’ or ‘very’ confident in the accuracy of coronavirus science, which is encouraging, but also raises questions about how to instil confidence in the sizeable remaining portion of the population. Finally, around 64% or respondents strongly agreed or tended to agree that public information is a true representation of coronavirus science, and those who did so were more likely to be older, male, and of white ethnicity.

These findings highlight the need to bolster accuracy in the reporting of science, which may in turn enhance other public perceptions, such as confidence in reports of science. They also highlight the need to enhance strategies of communication with younger people and with ethnic minorities. Further work is needed to understand if negative views relate to concerns about the trustworthiness of the information sources or in the science that underpins the public information.

## Strengths and limitations

A major strength of the dataset is that it was collected using interlocking quotas for key characteristics such as age, gender, and ethnicity to ensure a sample representative of adults living in England. We specified a minimum sample size of 2000, which is a standard one-day polling sample size from YouGov to both ensure the data was collected as concurrently as possible, and to achieve margins of error of 2–3 percentage points. Whilst some loss of data occurred when people declined to answer questions about key characteristics that were not specified as a representative quota, these losses did not result in marked changes to the overall precision of the dataset. For example, the margins of error for age and gender, with a complete sample size of 2025 (between 1.94% and 2.18%) were comparable to those of other characteristics with fewer samples, such as ‘coronavirus exposure’, N = 1791 (margins of error between 1.68% and 2.18%).

One unique aspect of our data collection is that it was conducted over the first nine days of the second national lockdown in November 2020. This meant that we captured views held during a time when participants might have been particularly aware of, focused on, and/or impacted by national restrictions and the coronavirus science that had been used to justify the policy decisions. We note that this was also before news of ‘variants of concern’ had emerged, and also before the release of information from clinical trials about vaccine efficacy. In the absence of a follow-up survey in more usual ‘newsworthy’ times, this remains an unquantified but potentially important influence on our findings.

To present these survey results, we grouped some of the responses across categories of respondents to make the data more usable and to improve statistical precision. This resulted in some loss of granularity, particularly for characteristics such as ethnicity, where many categories had to be combined. For example, only 1.0% of respondents identified as ‘mixed ethnicity’, and so we included this group in the ‘ethnic minority’ group, which itself accounted for only 12.1% of all respondents. We are aware that more resolution can be beneficial, and that there are conversations about identity and terminology that are relevant in such decisions, including in relation to the term ‘BAME’. Our method nonetheless followed the guidance about the reporting of ethnicity from the Office for National Statistics ( Office for National Statistics, 2015). We also combined those who had had a positive coronavirus test result with those who believed that they had had coronavirus, but who had not been tested, to explore people’s beliefs about coronavirus symptoms in relation to their trust and acceptance of the science. We note that community testing was not available in the UK in the early stages of the pandemic; therefore, to some extent, people’s belief that they had had coronavirus was more valid than a test result for questions about trust in March 2020. However, we acknowledge that we cannot be confident that those with symptoms would have volunteered for testing as the pandemic progressed and community testing became widely available.

A potential source of bias in our study is the use of recall to elicit views about coronavirus science at the start of the pandemic. We asked respondents to ‘think back’ to March 2020. Therefore, answers to the question about trust in coronavirus science in March 2020 are subject to recall and possibly various manifestations of social desirability bias. We were also dependent on the pool of registered YouGov respondents, as well as those who chose to self-report, which might not be completely representative of a random sample of adults in England, although specific quotas and weightings were used to adjust the data to be as representative as possible. 

We also observed that approximately 5–10% of responses to all of our survey questions were ‘don’t know’. This might reflect a lack of clarity in our phrasing of the questions, or a wider trend of understanding of the coronavirus pandemic in the public. We are unable to interpret this further without specific feedback from these individuals. Nevertheless, we report the data in the extended data and results section as an interesting outcome and reminder of the room for improvement in the communication of science ( Gooberman-Hill *et al.*, 2021b).

Finally, most of the questions in our study were cross-sectional, with some insight into how views had changed over time rendered through our questions about trust in March and November 2020. From these data, we aimed to present an overview of public views of science through descriptive statistics of categories of respondents of interest during the COVID-19 pandemic. We do not explore causation, or infer wider trends of opinions beyond those related to the coronavirus pandemic, nor infer longer-term trends in patterns of trust beyond that observed between March and November 2020. Further analyses would needed to identify and explore the effects of associations between key characteristics, including confounding effects in relation to the (crude) differences that we have presented here, all of which may be the subject of future analyses.

## Conclusion

In the coronavirus pandemic, rapid advances in current knowledge have been used to inform and explain policy decisions that impact on individuals and ask them to change their everyday behaviours, particularly those relating to the reduction or prevention of virus transmission. At the time of the study, scientists had used knowledge about the past or present to develop projections about the future, specifically in relation to infection, transmission, hospitalisation, and death rates. However, the uncertainty around the scenarios emerging from these models might not have been clearly communicated at key points during the pandemic, and understanding how this might have affected public views of science is of benefit to science communication.

Some public health measures in the coronavirus pandemic have been mandatory or unavoidable (for example, stay at home notices, lockdown measures, and closures of businesses and services), while others have been based on consensus and a voluntary adherence to guidance. However, the success of all measures and the avoidance of dissent depends on individual decisions about the appropriateness of the measures and whether adherence is possible or desirable. The relationship between trust and the use of measures to reduce transmission is complex, but in other contexts – such as the 2014–2015 Ebola outbreak in West Africa – use of precautions and uptake of government-recommended measures may have related more to degree of trust in government than the lack of understanding about disease transmission ( Blair *et al.*, 2017). Achieving success in transmission reduction measures depends on an agreement about the need for and value of such measures and, in the pandemic, science has been the key point of reference. Our study demonstrates that levels of trust appear high but can be improved, and that younger age and BAME ethnicity are associated with lower levels of trust in science and scientists’ work. Our study also indicates that trust in representations of science could improve and that there is scope to improve the extent to which uncertainty is conveyed. This would improve transparency ( Osman *et al.*, 2018), which has been shown not to reduce public trust in science or scientists ( Retzback & Maier, 2015; van der Bles *et al.*, 2019) and does not undermine trustworthiness ( Blastland *et al.*, 2020). There is a real chance that, communicated appropriately, improving transparency by including information about uncertainties could enhance trust both in science and in scientists. These may be important areas that enable greater and qualitatively improved public engagement with scientific evidence. This, in turn, could enable individuals’ decisions about their health behaviours to be more fully informed, and consequently, for adherence and agreement with measures to be enhanced. Finally, our study indicates that further research is needed to understand reasons for the associations, particularly those related to change in trust and views of science across age, ethnicity, and shielding, or other health status. Such work could make use of a variety of methodologies, including qualitative and quantitative approaches, and would help to inform public health and policy approaches in the future.

## Data Availability

University of Bristol Research Data Repository: Public views of covid science - Trust and uncertainty, https://doi.org/10.5523/bris.dexqujusyqvd2fxm8lxd2w321 ( Gooberman-Hill *et al.*, 2021a). Due to the nature of the third-party survey platform, consent to share data was not sought from research participants. Ethical approval was provided by the University of Bristol's Faculty of Health Sciences Research Ethics Committee for storage of the data on the University of Bristol Research Data repository. Requests for access will be directed to the Research Data team at Bristol and can be made via this form. The Research Data team has assessed the risk of re-identification of participants as low. Bona fide researchers can apply to access the data at this DOI, subject to a legally binding agreement. No authentic request for access will be refused and re-users will not be charged for any part of this process. University of Bristol Research Data Repository: Public views of covid science - Trust and uncertainty Tables, https://doi.org/10.5523/bris.3v4nw6rufyclh2kmv28ojrynm0 ( Gooberman-Hill *et al.*, 2021b). Data are available under the terms of the Non-Commercial Government License version 2.
