# Supplementary material for: Public views of coronavirus science and scientists: findings from a cross-sectional survey
Source: Wellcome Open Res. 2024 Dec 5;6:166. Originally published 2021 Jun 29. [Version 2] doi: 10.12688/wellcomeopenres.16780.2 (PMC11635929; doi:10.12688/wellcomeopenres.16780.2)
Supplement: Peer review history

# Reviewer report: Reviewer response for version 2

**DOI:** 10.21956/wellcomeopenres.25907.r114688

**Published:** 2025-03-27

**Authors:** Gillian Santorelli

**Affiliations:** 1 Bradford Institute for Health Research, Bradford, UK

The authors have amended the script accordingly.

Is the work clearly and accurately presented and does it cite the current literature?

Yes

If applicable, is the statistical analysis and its interpretation appropriate?

Not applicable

Are all the source data underlying the results available to ensure full reproducibility?

Yes

Is the study design appropriate and is the work technically sound?

Yes

Are the conclusions drawn adequately supported by the results?

Yes

Are sufficient details of methods and analysis provided to allow replication by others?

Yes

Reviewer Expertise:

Cohort studies, statistics, electronic health records.

I confirm that I have read this submission and believe that I have an appropriate level of expertise to confirm that it is of an acceptable scientific standard.

# Reviewer report: Reviewer response for version 2

**DOI:** 10.21956/wellcomeopenres.25907.r114689

**Published:** 2024-12-10

**Authors:** Matt Motta

**Affiliations:** 1 Boston University School of Public Health, Boston, MA, USA

I thank the authors for replying to my requests for revisions. I recognize now that the study's data collection Aims were primarily focused on providing descriptive data -- as a resource for other scholars -- and I appreciate the authors' willingness to clarify this throughout the article. Although I still think that data sharing and descriptive Aims might be best suited for some type of data descriptor article, I recognize that this is a somewhat unique situation. I'm happy to accept the paper for indexing without further revision.

Is the work clearly and accurately presented and does it cite the current literature?

Yes

If applicable, is the statistical analysis and its interpretation appropriate?

Not applicable

Are all the source data underlying the results available to ensure full reproducibility?

Yes

Is the study design appropriate and is the work technically sound?

Yes

Are the conclusions drawn adequately supported by the results?

Yes

Are sufficient details of methods and analysis provided to allow replication by others?

Yes

Reviewer Expertise:

Public Health; Health Policy; Health Politics; Political Science; Health Communication; Science Communication; Political Communication

I confirm that I have read this submission and believe that I have an appropriate level of expertise to confirm that it is of an acceptable scientific standard.

# Response

**Published:** 2024-12-13

**Authors:** Rachael Gooberman-Hill

**Affiliations:** University of Bristol, UK

The authors are enormously grateful to the reviewer for the thoughtful and helpful comments.

# Reviewer report: Reviewer response for version 1

**DOI:** 10.21956/wellcomeopenres.18508.r92774

**Published:** 2024-09-03

**Authors:** Gillian Santorelli

**Affiliations:** 1 Bradford Institute for Health Research, Bradford, UK

This article describes the level of public trust in scientists and scientific information during the Covid-19 pandemic. Data was obtained from two YouGov surveys completed by the general public at the beginning of the first and second lockdowns, approximately 8 months apart. 

 The article is interesting and well written. The methodology is clearly described and reproducible. The results section is rather long and would benefit from graphical presentations to allow the reader to visualise patterns in the data, both by participant characteristics and change over time. More use could be made of the data, e.g., regression analyses could be to identify predictors of mistrust, which is crucial for effective public health communication, yet this aspect has been overlooked in the current study. The study therefore misses an opportunity to provide a more nuanced analysis that could inform targeted strategies to build trust and improve the uptake of public health messages.

Is the work clearly and accurately presented and does it cite the current literature?

Yes

If applicable, is the statistical analysis and its interpretation appropriate?

Not applicable

Are all the source data underlying the results available to ensure full reproducibility?

Yes

Is the study design appropriate and is the work technically sound?

Yes

Are the conclusions drawn adequately supported by the results?

Yes

Are sufficient details of methods and analysis provided to allow replication by others?

Yes

Reviewer Expertise:

Cohort studies, statistics, electronic health records.

I confirm that I have read this submission and believe that I have an appropriate level of expertise to confirm that it is of an acceptable scientific standard, however I have significant reservations, as outlined above.

# Response

**Published:** 2024-11-29

**Authors:** Rachael Gooberman-Hill

**Affiliations:** University of Bristol, UK

**Authors’ response: Thank you.**

**We are pleased that the article was interesting and well written and that our description of the methods was satisfactory. As stated in the paper, strictly speaking we only conducted one survey including retrospectively seeking views about how respondents felt in March 2020. We agree with you that the results section is long. We have given this section further thought to see if there are areas that could be trimmed or represented in other ways. Currently, the article contains narrative description of the material accompanied by tabular displays and access to the full dataset in an open access repository.**

**In essence, the two key points made by Reviewer 3 reiterate the points raised by Reviewer 1 that we responded to about three years ago – first, that the Results section is lengthy and some detail could be replaced by graphical representations, and second, that multivariable analyses would be of value in identifying the statistically dominant ‘predictors’ of our three coronavirus-related outcomes (trust in scientific information, trust in scientists, and communication of uncertainty in scientific findings).**

**As noted previously, we agree that multivariable analyses would be of considerable value, but such aspects of research were from the start intended to be beyond the scope of our essentially descriptive paper. Moreover, such analyses would involve both non-trivial methodology (given the weighting that would ideally be involved in such analyses) and, as acknowledged by the reviewer, they could comprise not only analyses of data relating to each of the two time points but also considerations of factors associated with changes over time. The results of such regression analyses would therefore themselves require considerable scope in order to be presented in any useful detail, and far more so than could be yielded by shortening of the text in the existing Results section and/or replacing it by graphs. Hence, overall, this would require a very much longer – if admittedly potentially more insightful – article than the current version.**

**In summary, our response to these two (ultimately connected) points remains as before.**

**First, we prefer to retain the purpose and consequent limitations (already acknowledged in the paper) in this essentially descriptive article, and feel that the level of detail in the text is proportionate to its underlying objective. Graphs could be used as an alternative representational style but we would either lose the quantitative detail in the existing text or would be effectively duplicating information across the two representations.**

**Second, we already acknowledge that multivariable analyses would have been of value but they would in themselves have been extensive and would in any case have required the context of the present descriptive data (which amounts to univariable analyses) to be fully informative. We therefore hope that the paper, as presented, and the correspondingly available data set will form the basis for such future analyses by any researcher who wishes to undertake them.**

**Furthermore, we note that Reviewer 2 does acknowledge the value of our analyses and presentation, albeit with a recommendation for further details of conceptual motivation and clarity.**

**To improve clarity, we have made revisions to the Abstract (Background, Results and Conclusion), Introduction (paragraph 8), and Conclusion (paragraphs 1 and 2). **  

**Is the work clearly and accurately presented and does it cite the current literature?** Yes **Authors’ response: thank you.**  

**Is the study design appropriate and is the work technically sound?** Yes

**Authors’ response: thank you.**  

**Are sufficient details of methods and analysis provided to allow replication by others?** Yes

**Authors’ response: thank you.**  

**If applicable, is the statistical analysis and its interpretation appropriate?** Not applicable **Authors’ response: thank you.**  

**Are all the source data underlying the results available to ensure full reproducibility?** Yes

**Authors’ response: thank you.**  

**Are the conclusions drawn adequately supported by the results?** Yes

**Authors’ response: thank you.**

# Reviewer report: Reviewer response for version 1

**DOI:** 10.21956/wellcomeopenres.18508.r92775

**Published:** 2024-08-21

**Authors:** Matt Motta

**Affiliations:** 1 Boston University School of Public Health, Boston, MA, USA

This piece offers a descriptive and exploratory analysis of trust in scientific authorities in the United Kingdom during the COVID-19 pandemic. Drawing on insights from a nationally representative survey taking place in Fall 2020, the piece provides nationally representative insights into the degree to which different groups in society place trust in scientific authorities.

 In general, I found this piece to be well-written and substantively interesting. However, I have to confess that I found the piece to be somewhat lacking in conceptual motivation and clarity. Correspondingly, before I would be prepared to accept this piece for publication, I would encourage the authors to make several substantial revisions to the paper's front end.

 While I believe that descriptive and exploratory research has much to offer the literature on trust in science, I think that the authors can do much more to expand on *why *they are conducting this research.

 As the authors rightly note throughout the piece, a wealth of social science research has investigated the socio-demographic and political correlates of trust in scientific authorities.

 This, in my view raises an important question about the piece's conceptual motivation: if we already know a bit about why some people are more likely to trust health and scientific authorities than others, what exactly is the value offered by descriptive and exploratory research in this area? (I.e., because much of that description and exploration has, seemingly, already taken place).

 Consequently, at present, I think that the piece reads more like a "data descriptor" type of submission than it does a "research article" submission type.

 One way that the authors might consider re-framing the manuscript's "front end," in service of making the piece read more like a research article type of submission would be to tell the reader a bit more about *why *they expect to observe the demographic differences that they do. For example, why is it that people who test negative for COVID-19 or who take action to protect themselves and others from exposure to the virus are more likely to hold elevated trust in science? Is the idea that trust *facilitates *protective behavior (i.e., that people who take up these actions do so because they trust the public health recommendations made by scientific authorities)? Or, alternatively, does the relationship function in reverse; i.e., that those who take up the behavioral actions recommended by scientific experts, and who do not themselves contract COVID-19, become more likely to express trust in those issuing this guidance?

 I would encourage the authors to therefore amend the front end of the manuscript to (a) further clarify what exactly the field gains via indexing of this research (in addition to access to a quite useful and nationally representative dataset capturing this unique moment in time), and (b) offer additional analysis regarding why it is that they find what they expect to find.

Is the work clearly and accurately presented and does it cite the current literature?

Yes

If applicable, is the statistical analysis and its interpretation appropriate?

Not applicable

Are all the source data underlying the results available to ensure full reproducibility?

Yes

Is the study design appropriate and is the work technically sound?

Yes

Are the conclusions drawn adequately supported by the results?

Yes

Are sufficient details of methods and analysis provided to allow replication by others?

Yes

Reviewer Expertise:

Public Health; Health Policy; Health Politics; Political Science; Health Communication; Science Communication; Political Communication

I confirm that I have read this submission and believe that I have an appropriate level of expertise to confirm that it is of an acceptable scientific standard, however I have significant reservations, as outlined above.

# Response

**Published:** 2024-11-29

**Authors:** Rachael Gooberman-Hill

**Affiliations:** University of Bristol, UK

**Thank you for these interesting comments and for your view that our article provides a useful and nationally representative dataset. This was our ambition. Our response addresses the suggestion that we clarify the rationale for the work including the ‘conceptual motivation’.** **We have made revisions to the submitted article.**

**These can be found in the Abstract (Background, Results and Conclusion), the Introduction (paragraph 8) and Conclusion (paragraphs 1 and 2). Revisions clarify the study’s aims and give them greater prominence, as well as making future research opportunities more evident.**

**We explain that the study aimed to provide descriptive statistics and univariable associations between public views (of science, scientists and scientific uncertainty) and key variables (three drawn from previous studies and four from the multidisciplinary team’s expertise in relevant factors in the UK’s social and policy context at the time).**

**We have also clarified that further analysis of the datasets provided with this article are available should others wish to carry out multivariable analysis as a next step, which we also discuss in our response to Reviewer 3.**

**Some broader reflections on the reviewer’s comments relate to the overall aims and scope of our work.**

**Our study made available granular information about demographics and health status and views about science. While we were interested in providing analysis and data that can inform the development of interventions and communication approaches, we were also mindful that we should not go beyond the scope of our study’s aims nor make unwarranted claims based on the findings. We hope that our work provided findings that readers interested in health policy and science communication can use to inform thinking and further work, including future research in such (pandemic and emerging pandemic) situations.**

**Our work did signal the possibility that experience of health-related interventions and policies – such as the UK’s recommendation on ‘shielding’ – may be linked to views about science. Shielding was advised in 2020 by the UK Government as a means for people considered to be particularly at risk of harm from COVID-19 to protect their own health through self-regulated quarantine above and beyond national or regional ‘lockdown’ requirements. Other countries introduced similar policies, albeit with different terminology and recommendations – for instance in Ireland, ‘cocooning’ was the term of choice and recommendations were different to those in the UK. Since 2021, a body of literature has explored the health impact of such recommendations, including benefits and disbenefits. As the focus of our research was trust in science, and although we can see that people who were asked to shield appeared to be more likely to trust science than those who did not, it would have been beyond the bounds of our research to suggest reasons why those who were asked to shield appeared to be more likely (at population level) to trust science more than those not asked to shield. We were also unable to ascertain from the study whether those people who were asked to shield did so or not. There are a number of other (quantitative and qualitative) study designs that could be deployed to address cause and effect or mechanisms of action through which adherence to health advice may relate, if at all, to trust in science. Since the completion of our research in 2021, a number of studies have examined opinions of, or adherence to, policies and recommendations. These often apply qualitative approaches that deliver insights into patterns of and reasons for adherence.** **In addition, since the completion of our work, research by others has explored the relationship between behaviour and views about science. We note that such literature is increasingly attentive to understanding how misinformation and disinformation may have impact on public views. That work includes a focus on actions or interventions that may protect individuals and groups from the consequences of misinformation, for instance when such misinformation may lead to vaccine hesitancy or reduced confidence in public health interventions. This might reflect wider societal changes and concerns since 2021.  We remain interested in this field and continue to develop our work in this and related areas.**    

**Is the work clearly and accurately presented and does it cite the current literature?** Yes **Authors’ response: thank you.**

**Is the study design appropriate and is the work technically sound?** Yes

**Authors’ response: thank you.**  

**Are sufficient details of methods and analysis provided to allow replication by others?** Yes

**Authors’ response: thank you.**  

**If applicable, is the statistical analysis and its interpretation appropriate?** Not applicable **Authors’ response: thank you.**  

**Are all the source data underlying the results available to ensure full reproducibility?** Yes

**Authors’ response: thank you.**  

**Are the conclusions drawn adequately supported by the results?** Yes

**Authors’ response: thank you.**

# Reviewer report: Reviewer response for version 1

**DOI:** 10.21956/wellcomeopenres.18508.r45949

**Published:** 2021-09-21

**Authors:** Andreas S Papazoglou

**Affiliations:** 1 Aristotle University of Thessaloniki, Thessaloniki, Greece

The research article by Gooberman-Hill et al is a really interesting and significant contribution to the existing COVID-19-related literature. The manuscript reads well, despite the extensive description of the survey responses which render the manuscript long enough. The conclusions of the article are supported adequately by the results of the survey which was conducted following an appropriate and reproducible methodology.

 Aiming to potentially improve the quality of the conclusions drawn and raise the interest of the manuscript, I would suggest reducing the size of the current results section and further add a multivariate predictive model adjusted for the most relevant/important baseline variables (some of the following: age, gender, region, social grade, employment status, and parental status, ethnicity, marital status, education level, household size, house type, number of children in household, keyworker status, carer status, shielding status, and coronavirus exposure). This ordinal logistic regression model could be conducted in SPSS and could identify independent (baseline) predictors for the dependent ordinal outcomes of the study (Trust in scientific information, Trust in scientists to conduct accurate and reliable research, Communicating the uncertainty in coronavirus science,Confidence in the accuracy of coronavirus science, Is the public information a true representation of science?). For example, minority ethnicity and younger age could be detected as significant independent predictors of less trust in scientific information. Besides, it might be of interest to address similarly the ΔTrust, that is the difference between the first and second response [Trust(after)- Trust(before)]. Nevertheless, I understand that the above suggestions might alter too much the content of the manuscript and I do not know whether this is of the authors' interest. Hence I would just suggest the reduction of the size of the methods and results sections to an adequate minimum, so that the reader can easily read the whole manuscript. For instance, the authors could provide relevant figures (bar plots) with the survey response percentages and thereby there will be no need of extensive description on these results.

Is the work clearly and accurately presented and does it cite the current literature?

Yes

If applicable, is the statistical analysis and its interpretation appropriate?

Partly

Are all the source data underlying the results available to ensure full reproducibility?

Yes

Is the study design appropriate and is the work technically sound?

Partly

Are the conclusions drawn adequately supported by the results?

Yes

Are sufficient details of methods and analysis provided to allow replication by others?

Yes

Reviewer Expertise:

Public health, cardiovascular disease prevention

I confirm that I have read this submission and believe that I have an appropriate level of expertise to confirm that it is of an acceptable scientific standard, however I have significant reservations, as outlined above.

# Response

**Published:** 2023-06-12

**Authors:** Rachael Gooberman-Hill

**Affiliations:** University of Bristol, UK

Reviewer’s comment: The research article by Gooberman-Hill et al is a really interesting and significant contribution to the existing COVID-19-related literature. The manuscript reads well, despite the extensive description of the survey responses which render the manuscript long enough. The conclusions of the article are supported adequately by the results of the survey which was conducted following an appropriate and reproducible methodology.

**Authors’ response: We thank the reviewer for their kind words about our article. We were keen to make the information as comprehensive as possible.**

 Reviewer’s comment: Aiming to potentially improve the quality of the conclusions drawn and raise the interest of the manuscript, I would suggest reducing the size of the current results section and further add a multivariate predictive model adjusted for the most relevant/important baseline variables (some of the following: age, gender, region, social grade, employment status, and parental status, ethnicity, marital status, education level, household size, house type, number of children in household, keyworker status, carer status, shielding status, and coronavirus exposure). This ordinal logistic regression model could be conducted in SPSS and could identify independent (baseline) predictors for the dependent ordinal outcomes of the study (Trust in scientific information, Trust in scientists to conduct accurate and reliable research, Communicating the uncertainty in coronavirus science, Confidence in the accuracy of coronavirus science, Is the public information a true representation of science?). For example, minority ethnicity and younger age could be detected as significant independent predictors of less trust in scientific information. Besides, it might be of interest to address similarly the ΔTrust, that is the difference between the first and second response [Trust(after)- Trust(before)]. Nevertheless, I understand that the above suggestions might alter too much the content of the manuscript and I do not know whether this is of the authors' interest. Hence I would just suggest the reduction of the size of the methods and results sections to an adequate minimum, so that the reader can easily read the whole manuscript. For instance, the authors could provide relevant figures (bar plots) with the survey response percentages and thereby there will be no need of extensive description on these results. **Authors’ response: Thank you for this interesting thought. We have carefully considered whether to revise the presentation of the results and to reduce the methods section to enable this to happen. On reflection, we think it is in the interests of readers to maintain the detail and depth in the methods section so that our work is fully transparent, and to ensure that those interested can access the underlying dataset on the basis of full information about the work. We also considered multivariable analysis on the dataset and would think this the subject of a further manuscript as the aim of the material presented in this publication is to enable further work for those who wish to do so. The current article provides descriptive statistics from the study, identifies noteworthy findings, and provides access to the data.**

**Is the work clearly and accurately presented and does it cite the current literature? **Reviewer’s comment: Yes

**Authors’ response: Thank you.**

**Is the study design appropriate and is the work technically sound?** Reviewer’s comment: Partly

**Authors’ response: Thank you, we have responded to your suggestion relating to further analysis in the response above. We think that further analysis could reasonably be the subject of a further piece of work but is beyond the scope of this manuscript.**

**Are sufficient details of methods and analysis provided to allow replication by others? **Reviewer’s comment: Yes

**Authors’ response: Thank you.**

**If applicable, is the statistical analysis and its interpretation appropriate? **Reviewer’s comment: Partly

**Authors’ response: Thank you, we appreciate that a further analysis could be conducted with the available data, as described above we think this could be a good area for further work but we think it is unfortunately outside the scope of the current manuscript**.

**Are all the source data underlying the results available to ensure full reproducibility? **Reviewer’s comment: Yes

**Authors’ response: Thank you.**

**Are the conclusions drawn adequately supported by the results?** Reviewer’s comment: Yes

**Authors’ response: Thank you.**
